# Evaluation of the importance of ionic and osmotic components of salt stress on the photosynthetic efficiency of epiphytic lichens

**DOI:** 10.1007/s12298-022-01134-2

**Published:** 2022-02-03

**Authors:** Karolina Chowaniec, Kaja Rola

**Affiliations:** Faculty of Biology, Institute of Botany, Jagiellonian University, Gronostajowa 3, 30-387 Kraków, Poland

**Keywords:** Chlorophyll fluorescence, Ionic stress, Lichens, OJIP test, Osmotic stress, PSII maximal quantum yield

## Abstract

Salt stress can significantly disrupt the functioning of lichens which are self-sufficient symbiotic organisms inhabiting various severe environments. The aim was to test the effect of salt and sucrose on the photosynthetic efficiency of two selected epiphytic lichens inhabiting the interior of the land. Firstly, we compared the effect of salt and sucrose solutions of different concentrations. Secondly, the effect of salt and sucrose solutions with identical osmotic pressures was compared. The results showed that short-term salt stress leads to a significant reduction of *F*_*V*_*/F*_*M*_, greater changes in chlorophyll fluorescence parameters and OJIP transients compared to the osmotic effects induced by sucrose. This proved that the negative impact of salt stress is associated primarily with ionic effects. The most symptomatic effect of the ionic stress was a significant reduction of the utilisation of trapped energy in electron transport and thereby down-regulation of electron transfer. Since lichens are resistant to a temporary lack of water, ionic stress could have more serious consequences than osmotic stress itself. *Hypogymnia physodes* was more sensitive to salt stress than *Pseudevernia furfuracea*, but the reduction of photosynthetic efficiency was not permanent since after 24 h *F*_*V*_*/F*_*M*_ returned to the level characteristic for healthy lichens. Nevertheless, repeated exposure to salt may reduce the vitality of lichens growing along communication routes sprinkled with salt in the winter season. Finally, the changes in certain JIP-test parameters were stronger than *F*_*V*_*/F*_*M*_, thus they could be better indicators of salt stress in lichens.

**Supplementary Information:**

The online version contains supplementary material available at 10.1007/s12298-022-01134-2.

## Introduction

Lichens are self-sufficient organisms that constitute a symbiotic relationship between a heterotrophic fungus and autotrophic algae and/or cyanobacteria. Due to the lack of root systems and protective cuticles they are especially sensitive to various pollutants and toxic substances (Tyler 1989). This physiological nature of lichens causes those substances present in a surrounding environment easily absorbed through the whole surface of the thalli (Nimis et al. 2002). Although lichens are sensitive to various pollutants and consequently have widely been used in biomonitoring studies, they are able to inhabit the most severe environments on Earth, from Antarctica to dry and hot deserts (Grube and Blaha 2005). Examples of such extreme areas are saline environments such as sea shores, dry areas such as deserts, semi-deserts, and steppes, as well as the immediate vicinity of roads where salt is used for snow removal.

In many areas of the world salt stress is an important factor that disrupts the functioning of organisms such as plants and lichens, and severely limits crops (Nash III and Lange 1988). The excess of salt in the environment has been one of the major concerns in recent years. Elevated salt concentrations lead to a decrease in hydric potential affecting water availability in lichens (Hasegawa et al. 2000). One of the main consequences of salt stress is the loss of intracellular water. Water loss is a dynamic phenomenon and certain acclimation can occur in lichens (Lange and Green 2005). Nevertheless, the accumulation of saline elements in thalli may induce both strong dehydration in lichens, but also ionic imbalances, and a deep loss in net photosynthesis of lichen photobionts (Matos et al. 2011). Moreover, high salinity induces hyperosmotic shock that generates oxidative stress and causes various metabolic disorders (Erdman and Hagemann 2001; Delmail et al. 2013). Therefore, lichens and especially photobionts associated with them are exposed to severe stress due to changes induced by increased salinity. Although several papers have attempted to assess the effect of salt stress on lichen photobionts, separating the effects of osmotic and ionic components on photosynthesis in lichens remains largely unexplored (see also Pan et al. 2021).

Lichens are poikilohydric organisms, so their hydration depends on the water content in the environment in which they grow (Honegger 2007). The exposure to dehydration leads to a gradual loss of photosynthetic activities of the photosynthetic partner, as evidenced by changes in the photosystem II (PSII) and is manifested by a decrease in the efficiency of transfer of the energy absorbed by it. The reduction of the maximum efficiency of PSII can lead to irreversible damage within it and consequently viability reduction of the whole thallus (Matos et al. 2011). The rate of respiration is also slowed down as a result of the gradual loss of pressure potential in the cells (Hajek et al. 2006). The induced osmotic shock, ionic imbalances, and the production of reactive oxygen species in lichen thallus can also contribute to disturbances in the integrity of cell membranes, damage to the cytoskeleton, or a reduction of protein or enzyme activity (Delmail et al. 2013; Yemets et al. 2015).

Lichens have developed many mechanisms that protect them against the negative effects of salinity (e.g. Grube and Blaha 2005). An example is the presence of an antioxidant system that protects the thallus from ROS, which can cause many cellular disturbances such as changes in the structure of enzymes involved in important metabolic pathways (Kranner et al. 2005; Delmail et al. 2013). In response to salinity, lichens produce specific metabolites called ‘osmolytes’ that serve as protection of proteins and are involved in maintaining continued water influx. Thanks to these substances, lowering of the internal osmotic potential is possible (Grube and Blaha 2005). Moreover, they could decrease the negative impact of ions on enzyme activity. Examples of this type of compounds include sucrose, fructose, glycerol, or trehalose (Hasegawa et al. 2000). Finally, microorganisms inhabiting lichen thallus may play a role in protecting the thallus against the influence of salinity, demonstrating the ability to store substances in the cytoplasm (e.g. trehalose or amino acids such as alanine) that protect against osmotic shock (Delmail et al. 2013).

One of the sources of salt in urban and rural environments is road salting used for safety precautions on the  roads during winter seasons for the prevention of ice formation on the road surface, snow accumulation, and re-freezing processes. Dissolved salt in melted water turn into airborne droplets by passing vehicles and sprayed away from the road. Moreover, aerial dispersion of salt comes from dry salt getting kicked up by tires and salty melted water. Various epiphytic lichens growing on trees along the roads are constantly exposed to salt stress. Although the resistance and adaptations at the morphological, anatomical, and biochemical levels of certain salt-resistant, coastal species have been recognized (see Delmail et al. 2013), there is still little information on species that naturally inhabit the interior of the land. Recognizing the effect of salt of anthropogenic origin on photosynthesis in various inland lichen species can help in the actual assessment of its effect on the physiology of these organisms.

The aim of this study was to test the effect of salt (solutions of different NaCl concentrations) and sucrose (solutions of different C_12_H_22_O_11_ concentrations) on chlorophyll fluorescence parameters in two selected epiphytic lichens. In the first stage we compared the effect of salt and sucrose solutions on photosynthetic efficiency through chlorophyll fluorescence measurements taking into account of experimental groups representing solutions of different concentrations. Since sucrose solutions cause only disturbances in the osmotic balance of cells, the effect of salt solutions additionally induces a negative effect due to the presence of sodium and chlorine ions. In another experiment, we tested the effect of salt and sucrose solutions with identical osmotic pressures on photosynthetic efficiency. This was tested to verify whether the salt treatment  will have  greater effects as compared to sucrose and further to estimate the influence of osmotic and ionic components of salt stress on photosynthetic efficiency.

## Materials and methods

### Selected species

*Pseudevernia furfuracea* (L.) Zopf. is a cosmopolitan epiphytic species. It has a flattened subfruticose thallus and reproduces vegetatively by isidia, which are produced on the upper surface of the thallus (Nimis 2016). The species is associated with photobionts representing green algae of the genus *Trebouxia* (Ahmadjian 1993). *Pseudevernia furfuracea* is commonly used as a biomonitor of airborne trace elements and other pollutants (Malaspina et al. 2014a; Incerti et al. 2017) and is widely utilised in bioindication studies assessing the degree of environmental pollution based on the changes of its physiological condition (Malaspina et al. 2018).

*Hypogymnia physodes* (L.) Nyl. is a widespread epiphytic lichen with foliose thallus. It reproduces asexually by white powdery soredia formed under the outer thallus edges. The photobiont is represented by a green alga of the genus *Trebouxia* (Nimis 2016). *Hypogymnia physodes* is also commonly used in biomonitoring studies due to its ability to accumulate various trace elements such as lead or copper (e.g. Rusu et al. 2006).

### Sample collection and handling

*Pseudevernia furfuracea* and *H. physodes* were collected in 2020 from living and fallen trees in a mid-forest clearing in Podsarnie (49°32′2′′N; 19°46′3′′E, 786 m.s.l.), located in the Orawsko-Podhalański Beskids, which is a range of Beskid Żywiecki Mts. (S Poland; Fig. S1). The data provided by the nearest air pollution measuring station indicate good air quality in the sampling area (data obtained from Voivodeship Inspectorate for Environmental Protection in Kraków). Freshly collected thalli were packed into paper bags and transported to the Soil Chemical Laboratory of the Institute of Botany of the Jagiellonian University.

## Experimental design

Before each experiment, lichen thalli were kept for 24 h in a chamber with 95% relative humidity. This step is essential to reactivate physiological activity and to re-establish the integrity of cellular membranes (Buck and Brown 1979). The experiment was performed under normal laboratory light which varied from 55 to a maximum of 100 μmol m^−2^ s^−1^ at room temperature of 20 °C. In the first stage of the experiment pure salt solutions (NaCl) with the following concentrations: 0.9, 1.8, 2.2, 2.8, 3.5, 3.9 M and pure sucrose solutions with the following concentrations: 0.15, 0.30, 0.35, 0.45, 0.55, 0.60 M were prepared. The 0 M solution in each group was treated as a control group and constituted of distilled water. In the second stage of the experiment, 0.87 M salt solution and 1.62 M sucrose solution were used; the solutions were characterised by the same osmotic pressure (π = 39 504 hPa). As a result, it was possible to directly compare the effects of ionic (NaCl) and non-ionic (sucrose) stress induced by solutions characterised by the same osmotic pressure. The thalli of *P. furfuracea* and *H. physodes* were then transferred to vessels with appropriate solutions and shaken on a vibrating shaker (Vibramax 100, Heidolph) for 2 h (rotation speed 300 rpm). Then, the thalli were transferred to a climatic chamber (approx. 95% relative humidity). After 1 h, the chlorophyll fluorescence was measured in 8 replications for each experimental group (the first day of the experiment). Then the thalli were left in the climatic chamber and after 24 h the measurements were taken again (the second day of the experiment).

### Fluorescence measurements

Chlorophyll fluorescence measurements were performed using Handy-PEA fluorimeter (Plant Efficiency Analyzer, Hansatech Instruments Ltd, Norfolk, England). Lichen samples were dark-adapted for 20 min before measurements. Chlorophyll fluorescence was induced by red light (wavelength 650 nm) provided by an array of high-intensity LEDs. Data were recorded after a saturating light pulse (2400 µmol/m^2^/s). All the fluorescence transients were recorded with a time span from 10 μs to 1 s.

The vitality of the lichen photobiont was assessed by the maximum quantum yield of PSII photochemistry in the dark-adapted state: *F*_*V*_*/F*_*M*_ = (*F*_*M*_ – *F*_*0*_)/*F*_*M*_, where *F*_*0*_ and *F*_*M*_ are minimal and maximal Chl *a* fluorescence intensity and *F*_*V*_ = (*F*_*M*_ – *F*_*0*_) is the variable fluorescence. The *F*_*0*_ parameter constituted the value calculated using extrapolation back to time point 0 using least squares regression of the first few data points. The *F*_*V*_*/F*_*M*_ values corresponding to healthy lichens were considered to be > 0.6 for chlorolichens (after Jensen and Kricke 2002). For a more precise analysis of the impact of stress on studied lichens, other parameters of chlorophyll fluorescence were analysed (Table 1).

**Table 1:** The selected JIP-test parameters calculated on the basis of fast fluorescence kinetics used in further analyses

Fluorescence parameter	Description
*Basic*	
*F*_*0*_	Minimal fluorescence intensity
*F*_*V*_*/F*_*M*_	Maximum quantum yield of PSII photochemistry
*The specific energy fluxes per reaction centre (RC)*	
ABS/RC	Specific absorption flux per reaction centre
DI_0_/RC	Dissipated energy flux per reaction centre
TR_0_/RC	Trapped energy flux per reaction centre
ET_0_/RC	Electron transport flux per reaction centre
*Quantum yields and efficiencies*	
Phi (P_0_)	Probability that an absorbed photon will be trapped by the reaction centre of PSII
Psi (E_0_)	Probability that a trapped exciton moves an electron into the electron transport chain beyond QA
Phi (E_0_)	Quantum yield of electron transport
Phi (R_0_)	Quantum yield of reduction of end electron acceptors at the PS I acceptor side
*Phenomenological energy fluxes per excited cross-section (CS)*	
ABS/CS	Specific absorption flux per excited cross-section
DI_0_/CS	Dissipated energy flux per excited cross-section
TR_0_/CS	Trapped energy flux per excited cross-section
ET_0_/CS	Electron transport flux per excited cross-section
*Performance index*	
PI_ABS_	Performance index (potential) for energy conservation from photons absorbed by PSII to the reduction of intersystem electron acceptors

### Data analysis

With respect to the first stage of the experiment three main qualitative predictors were distinguished: ‘Concentration’: for salt: 0, 0.9, 1.8, 2.2, 2.8, 3.5, 3.9 M and for sucrose: 0, 0.15, 0.30, 0.35, 0.45, 0.55, 0.60 M, ‘Day’: day 1, day 2 and ‘Species’: *Pseudevernia furfuracea*, *Hypogymnia physodes*. The changes in the *F*_*V*_*/F*_*M*_ parameter, which determines the maximum efficiency of PSII, were analysed in detail. Two-way analyses of variance (two-way ANOVA; *P* < 0.05) were performed to assess the effect of (1) ‘Concentration’ and ‘Day’ on the *F*_*V*_*/F*_*M*_ parameter for salt and sucrose solutions; separately for each species; (2) ‘Concentration’ and ‘Species’ on the *F*_*V*_*/F*_*M*_ parameter for salt and sucrose solutions; separately for each day of the experiment. Then, the significance of differences between particular experimental groups was verified with Tukey’s HSD post-hoc tests (*P* < 0.05). The significance of differences in terms of photosynthetic parameters characterising PSII functionality between particular salt and sucrose treatments on the first day of the experiment was tested with one-way analyses of variance (*P* < 0.05) followed by Tukey’s HSD post-hoc tests for both lichen species, separately. Before performing these analyses, the normality distribution in particular groups was checked using the Kolmogorov–Smirnov test. Levene's test was used to verify the homogeneity of variances. Box-Cox transformation was applied if necessary.

As regards the second stage of the experiment, the obtained values of photosynthetic parameters characterising PSII functionality for 0.87 M salt solution and 1.62 M sucrose solution were converted in relation to the control values. The mean value for the control samples (*n *= 8) was adopted for calculations. Then, Student's t-tests (*P* < 0.05) were used to verify the significance of differences between salt and sucrose solutions for each species and day of the experiment, separately. Next, Student's t-tests (*P* < 0.05) were applied to test the significance of differences in photosynthetic parameters characterising PSII functionality between 0.87 M salt solution and control as well as for 1.62 M sucrose solution and control treatment, for each species and day of experiment, separately. Prior to the analysis, the normality distribution o was checked using the Kolmogorov–Smirnov test and Levene's test was used to verify homogeneity of variances. Box-Cox transformation was applied if necessary. Statistical analyses were performed in the STATISTICA 12 (StatSoft, Tulsa, OK).

For both stages of the experiment the chlorophyll fluorescence induction curves were plotted for particular experimental groups (averaged values for *n* = 8 measurements) including the division into species and days of the experiment. This was based on the results of chlorophyll fluorescence signals at short time intervals, starting from 10 μs, and ending before 1 s. The fast fluorescence kinetic outlines a transient curve plotted on a log-time axis to visualise a sequence of steps called OJIP (Strasser et al. 2000).

Regarding the results of the first stage of the experiment, spider plots were created to illustrate the effect of salt and sucrose stress on various parameters characterising PSII functionality on the first day of the experiment, for each species separately. The graphs were used to verify if there are more sensitive parameters than *F*_*V*_*/F*_*M*_ that could indicate the effect of stress. The plots were based on the values normalised to the control treatment, enabling the comparison of parameters measured on different scales.

## Results

### Impact of different salt and sucrose treatments on photosynthetic efficiency during the first stage of the experiment

**Fig. 1:**
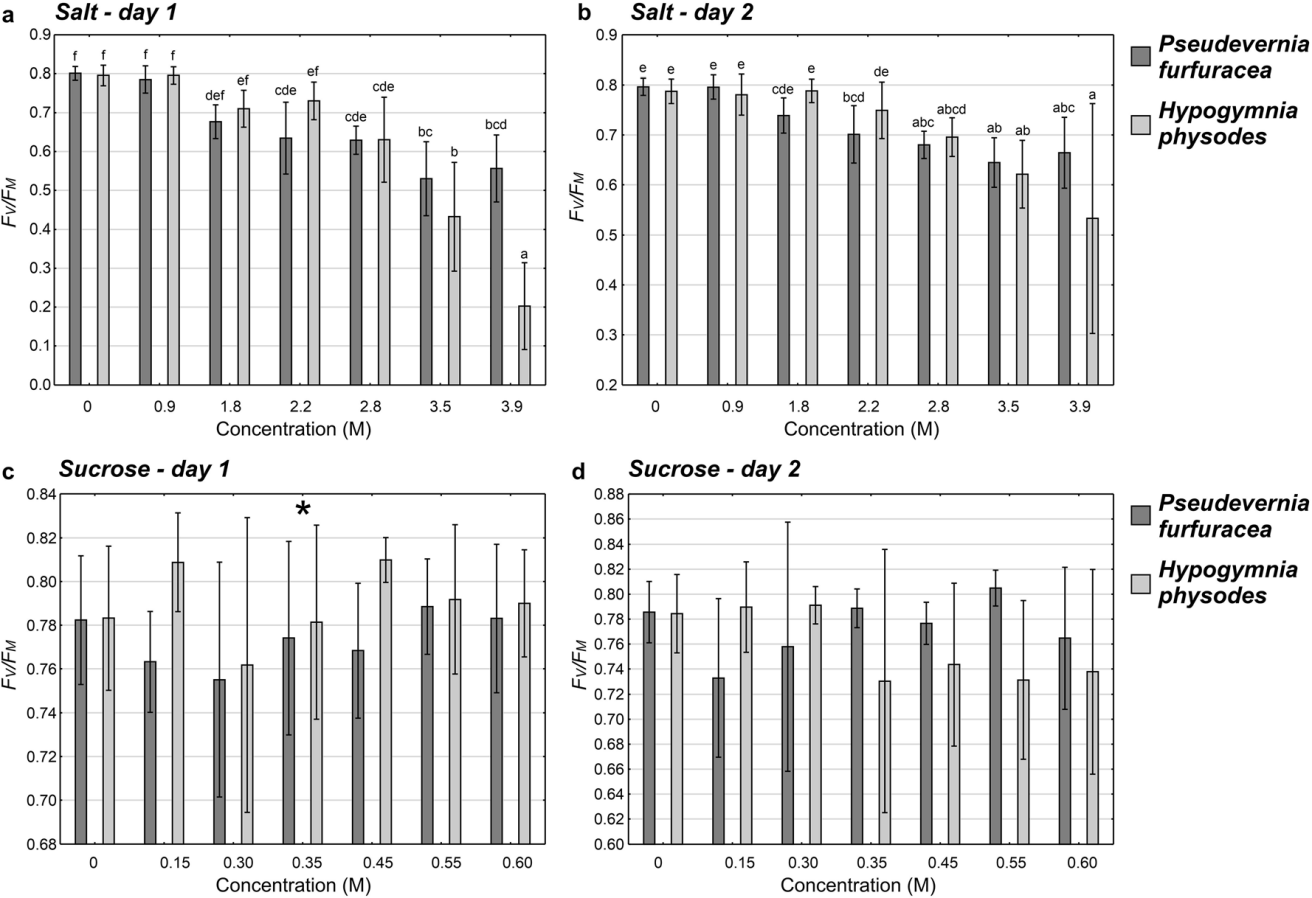
The *F*_*V*_*/F*_*M*_ parameter in particular experimental groups (means ± SE; *n* = 8) for different concentrations of salt (**a, b**) and sucrose (**c, d**) solutions in two subsequent days of the experiment for *Hypogymnia physodes* and *Pseudevernia furfuracea*. The different letters above the bars indicate statistically significant differences (*P* < 0.05). Lowercase letters indicate a statistically significant interaction between lichen species and solution concentration. The asterisk indicates the significant main effect of species. See Table S1 for details on the main effects and interactions

Comparison of the simultaneous effect of ‘Concentration’ and ‘Species’ on the *F*_*V*_*/F*_*M*_ parameter for salt solutions showed a significant interaction between these independent variables on both days of the experiment (Table S1). Significantly the lowest values of *F*_*V*_*/F*_*M*_ were recorded for solutions with a concentration of 3.9 M in the case of *H. physodes* (Fig. 1a, b). When the effect of sucrose on the *F*_*V*_*/F*_*M*_ parameter was analysed for the first day of the experiment, no significant differences between particular concentrations were noted, while the effect of ‘Species’ proved to be significant (Table S1, Fig. 1c). For each concentration, the *F*_*V*_*/F*_*M*_ values were lower in the case of *P. furfuracea*. On the second day of the experiment a significant interaction between ‘Species’ and ‘Concentration’ was recorded, but the post-hoc test did not show significant differences (Table S1, Fig. 1d).

In *H. physodes*, the *F*_*V*_*/F*_*M*_ parameter was influenced by both the ‘Day’ and ‘Concentration’ of salt solution (significant ‘Day’ × ‘Concentration’ interaction; Table S1). This parameter was significantly lower on the first day of the experiment for the 3.9 M concentration compared to the remaining concentrations in two consecutive days of the experiment, with the exception of the 3.5 M concentration on the first day of the experiment (Fig. S2a). No significant differences were found between particular sucrose concentrations, while the effect of ‘Day’ proved to be significant (Table S1, Fig. S2b). Analysing the effect of salt concentration and the day of experiment on the *F*_*V*_*/F*_*M*_ parameter in *P. furfuracea*, the interaction between ‘Concentration’ and ‘Day’ turned out to be significant (Table S1, Fig. S2c). Significantly lowest *F*_*V*_*/F*_*M*_ values were recorded for the concentration of 3.5 M on the first day of the experiment; they differed significantly from all other concentrations on both days of the experiment, except for the concentration of 3.9 M on the first day of the experiment. Concerning the effect of sucrose, significant differences between particular concentrations were noted, regardless of the experiment day (Table S1, Fig. S2d). The *F*_*V*_*/F*_*M*_ parameter was significantly lower after treatment with 0.15 M solution compared to 0.55 M solution.

The salt stress caused a significant increase in the values of the ABS/RC, DI_0_/RC, and DI_0_/CS parameters in *H. physodes* on the first day of the experiment and a significant decrease of the *F*_*0*_, *F*_*V*_*/F*_*M*_, TR_0_/RC, ET_0_/RC, Phi (P_0_), Psi (E_0_), Phi (E_0_), Phi (R_0_), ABS/CS, TR_0_/CS, ET_0_/CS, PI_ABS_ parameters versus control (Table 2). This effect was particularly noticeable for 3.5 M and 3.9 M solutions (Fig. S3a). In the case of *P. furfuracea*, the results were similar, except for *F*_*0*_ and ABS/CS the values of which were significantly increased in all experimental groups compared to the control (Fig. S3b). Significant differences were recorded for all parameters except for TR_0_/CS (Table 2). The changes in chlorophyll fluorescence parameters after treatment with sucrose solutions did not differ as considerably from the control compared to the effect of salt solutions (Fig. S4). There were no significant differences in the analysed parameters between any of the sucrose treatments and the control for *H. physodes* (one-way ANOVA, *P* > 0.05; data not shown). As regard *P. furfuracea*, the stress induced by sucrose caused significant changes between control and single experimental groups of only 5 out of 15 tested parameters, i.e. *F*_*0*_, TR_0_/RC, ABS/CS, TR_0_/CS, ET_0_/CS (Fig. S4b).

**Table 2:** Photosynthetic parameters characterising PSII functionality (means ± SE) in *Pseudevernia furfuracea* and *Hypogymnia physodes* after treatment with salt solutions on the first day of the experiment

Parameter	*Pseudevernia furfuracea*							F	P
	0 M (control)	0.9 M	1.8 M	2.2 M	2.8 M	3.5 M	3.9 M		
*F*_*0*_	428.13 ± 26.39a	496.38 ± 39.17ab	585.50 ± 24.56ab	628.50 ± 63.61b	562.50 ± 53.19ab	678.75 ± 40.68b	650.38 ± 32.75b	4.64	0.001
*F*_*V*_*/F*_*M*_	0.80 ± 0.01d	0.79 ± 0.01d	0.68 ± 0.02c	0.63 ± 0.03bc	0.63 ± 0.01abc	0.53 ± 0.03a	0.56 ± 0.03ab	31.41	< 0.001
ABS/RC	3.28 ± 0.07ab	3.22 ± 0.11a	3.78 ± 0.15bcd	3.84 ± 0.29bcd	3.53 ± 0.08abc	4.61 ± 0.38d	4.39 ± 0.25 cd	9.38	< 0.001
DI_0_/RC	0.65 ± 0.03a	0.70 ± 0.06a	1.23 ± 0.09b	1.46 ± 0.25bc	1.31 ± 0.06bc	2.25 ± 0.38c	2.00 ± 0.24bc	28.28	< 0.001
TR_0_/RC	2.63 ± 0.05c	2.52 ± 0.06bc	2.55 ± 0.09bc	2.38 ± 0.07abc	2.22 ± 0.06a	2.36 ± 0.05ab	2.39 ± 0.03abc	5.01	< 0.001
ET_0_/RC	0.90 ± 0.02d	0.78 ± 0.02 cd	0.74 ± 0.04c	0.48 ± 0.02ab	0.53 ± 0.03b	0.39 ± 0.04ab	0.35 ± 0.05a	42.05	< 0.001
Phi (P_0_)	0.80 ± 0.01c	0.79 ± 0.01c	0.68 ± 0.02b	0.63 ± 0.03ab	0.63 ± 0.01ab	0.53 ± 0.03a	0.56 ± 0.03a	31.56	< 0.001
Psi (E_0_)	0.34 ± 0.01d	0.31 ± 0.01 cd	0.29 ± 0.01c	0.20 ± 0.01ab	0.24 ± 0.01b	0.16 ± 0.01a	0.15 ± 0.02a	42.91	< 0.001
Phi (E_0_)	0.27 ± 0.01d	0.24 ± 0.01d	0.20 ± 0.01c	0.13 ± 0.01ab	0.15 ± 0.01b	0.09 ± 0.01a	0.09 ± 0.02a	52.82	< 0.001
Phi (R_0_)	0.09 ± 0.00d	0.07 ± 0.00c	0.06 ± 0.00bc	0.03 ± 0.00a	0.05 ± 0.00b	0.03 ± 0.00a	0.03 ± 0.01a	40.39	< 0.001
ABS/CS	428.13 ± 26.39a	496.38 ± 39.17ab	585.50 ± 24.56ab	628.50 ± 63.61b	562.50 ± 53.19ab	678.75 ± 40.68b	650.38 ± 32.75b	4.64	0.001
DI_0_/CS	84.43 ± 4.34a	109.31 ± 15.48a	189.20 ± 11.92b	219.60 ± 17.98bcd	206.34 ± 18.48bc	324.77 ± 42.31d	288.75 ± 24.31 cd	32.07	< 0.001
TR_0_/CS	343.70 ± 22.65	387.06 ± 24.22	396.30 ± 19.24	408.90 ± 52.52	356.16 ± 36.91	353.98 ± 22.12	361.62 ± 27.75	0.62	0.709
ET_0_/CS	117.42 ± 8.13b	118.85 ± 5.68b	114.86 ± 6.14b	83.38 ± 12.27ab	85.96 ± 11.72ab	58.78 ± 7.24a	54.38 ± 9.36a	9.31	< 0.001
PI_ABS_	0.65 ± 0.04d	0.55 ± 0.07d	0.24 ± 0.03c	0.14 ± 0.03ab	0.15 ± 0.01bc	0.06 ± 0.01a	0.07 ± 0.02ab	47.76	< 0.001

Parameter	*Hypogymnia physodes*							F	P
	0 M (control)	0.9 M	1.8 M	2.2 M	2.8 M	3.5 M	3.9 M		
*F*_*0*_	606.75 ± 70.35b	532.00 ± 34.55b	591.63 ± 37.93b	528.50 ± 23.43b	611.13 ± 43.10b	571.50 ± 33.98b	323.13 ± 71.37a	4.59	0.001
*F*_*V*_*/F*_*M*_	0.80 ± 0.01d	0.80 ± 0.01d	0.71 ± 0.02bc	0.73 ± 0.02 cd	0.63 ± 0.04b	0.43 ± 0.05a	0.20 ± 0.04a	123.01	< 0.001
ABS/RC	3.19 ± 0.12a	3.14 ± 0.14a	3.73 ± 0.19ab	3.19 ± 0.13a	4.11 ± 0.28bc	5.42 ± 0.50c	12.03 ± 2.03d	27.71	< 0.001
DI_0_/RC	0.65 ± 0.04a	0.64 ± 0.04a	1.10 ± 0.12bc	0.87 ± 0.09ab	1.59 ± 0.27c	3.23 ± 0.53d	10.08 ± 2.06e	68.84	< 0.001
TR_0_/RC	2.54 ± 0.10b	2.50 ± 0.11b	2.63 ± 0.08b	2.32 ± 0.06ab	2.51 ± 0.04b	2.19 ± 0.11ab	1.95 ± 0.10a	7.10	< 0.001
ET_0_/RC	0.96 ± 0.03d	0.88 ± 0.02d	0.74 ± 0.03c	0.64 ± 0.02c	0.46 ± 0.05b	0.25 ± 0.02a	0.27 ± 0.03a	105.83	< 0.001
Phi (P_0_)	0.80 ± 0.01d	0.80 ± 0.01d	0.71 ± 0.02 cd	0.73 ± 0.02d	0.63 ± 0.04c	0.43 ± 0.05b	0.20 ± 0.04a	122.97	< 0.001
Psi (E_0_)	0.38 ± 0.02d	0.35 ± 0.01d	0.28 ± 0.01c	0.28 ± 0.01c	0.19 ± 0.02b	0.12 ± 0.01a	0.14 ± 0.02ab	48.56	< 0.001
Phi (E_0_)	0.30 ± 0.01d	0.28 ± 0.01d	0.20 ± 0.01c	0.20 ± 0.01c	0.12 ± 0.02b	0.05 ± 0.01a	0.03 ± 0.00a	258.07	< 0.001
Phi (R_0_)	0.09 ± 0.02d	0.10 ± 0.01d	0.07 ± 0.00 cd	0.06 ± 0.00 cd	0.04 ± 0.00bc	0.02 ± 0.00ab	0.01 ± 0.00a	27.22	< 0.001
ABS/CS	606.75 ± 70.35b	532.00 ± 34.55b	591.63 ± 37.93b	528.50 ± 23.43b	611.13 ± 43.10b	571.50 ± 33.98b	323.13 ± 71.37a	4.59	0.001
DI_0_/CS	127.27 ± 21.59a	109.65 ± 9.93a	171.23 ± 15.34ab	141.51 ± 8.79ab	216.96 ± 14.78bc	316.00 ± 21.51c	238.47 ± 41.21bc	21.61	< 0.001
TR_0_/CS	479.48 ± 49.57c	422.35 ± 25.66c	420.40 ± 29.19c	386.99 ± 22.00bc	394.16 ± 47.62bc	255.50 ± 40.96b	84.65 ± 32.92a	17.11	< 0.001
ET_0_/CS	187.67 ± 29.51d	150.40 ± 12.27d	120.14 ± 11.84 cd	108.85 ± 9.55 cd	78.35 ± 15.33c	31.07 ± 6.04b	8.87 ± 2.43a	38.97	< 0.001
PI_ABS_	0.77 ± 0.05d	0.71 ± 0.07d	0.29 ± 0.04c	0.36 ± 0.05c	0.14 ± 0.04b	0.03 ± 0.01a	0.00 ± 0.00a	195.46	< 0.001

The results of the one-way analysis of variance are provided in the table. The different letters next to means indicate statistically significant differences (*P* < 0.05) between particular salt treatments according to post-hoc Tukey’s tests

### Impact of salt and sucrose solutions with the same osmotic pressure on photosynthetic efficiency during the second stage of the experiment

Significant differences in *F*_*V*_*/F*_*M*_ expressed as a percentage of the control were observed between salt and sucrose solutions with the same osmotic pressure only in the case of *H. physodes* on the first day of the experiment (Student t-test, *P* < 0.05; Table 3). For all species and experimental days, the mean values were also always lower for salt compared to the sucrose solution, but these differences were not significant. Regarding other fluorescence parameters, significant differences between the salt and sugar treatment effect in relation to the control were noted for ET_0_/RC, Psi (E_0_), Phi (E_0_), Phi (R_0_) for *P. furfuracea* on the first day of the experiment. Each of these parameters was decreased in the salt-treated thalli relative to the control, unlike the sugar-treated samples, the values of which oscillated around the control values (Student t-test, *P* < 0.05; Table 3). As regard *H. physodes*, significant differences in salt and sugar effects relative to the control were recorded for the following parameters: *F*_*0*_, ET_0_/RC, Phi (P_0_), Psi (E_0_), Phi (E_0_), ABS/CS, TR_0_/CS, ET_0_/CS, and PI_ABS_, which values were significantly lower for salt-treated as compared to sugar-treated thalli (Student t-test, *P* < 0.05; Table 3). The induced disturbances were not permanent, because for both species on the second day of the experiment, no significant differences were noted (Table 3). Comparing the changes caused by the studied salt and sucrose solutions on the parameters of chlorophyll fluorescence in relation to the corresponding control values, significant reductions of ET_0_/RC, Psi (E_0_), Phi (E_0_), and Phi (R_0_) in *P. furfuracea* and ET_0_/RC in *H. physodes* were observed (Student t-test, *P* < 0.05). On the other hand, sucrose treatment did not caused any significant changes in chlorophyll fluorescence parameters compared to the control (Table S2).

**Table 3:** Comparison of various photosynthetic parameters characterising PSII functionality (expressed as a percentage of the control; means ± SD) in the species *Pseudevernia furfuracea* and *Hypogymnia physodes* after treatment with salt (0.87 M) and sucrose (1.62 M) solutions of the same osmotic pressure on the first and the second day of the experiment

Parameter	Day of experiment	*Pseudevernia furfuracea*				*Hypogymnia physodes*			
		Salt	Sucrose	t	P	Salt	Sucrose	t	P
*F*_*0*_	Day 1	115.9 ± 25.9	102.2 ± 21.3	1.16	0.265	**87.7 ± 16.1**	**113.1 ± 13.6**	− **3.42**	**0.004**
	Day 2	106.3 ± 17.7	102.8 ± 15.6	0.42	0.680	91.0 ± 16.1	88.8 ± 6.8	0.35	0.732
*F*_*V*_*/F*_*M*_	Day 1	98.0 ± 4.3	99.7 ± 3.0	− 0.91	0.378	**100.0 ± 2.8**	**104.2 ± 2.4**	− **3.25**	**0.006**
	Day 2	99.9 ± 3.0	99.8 ± 1.2	0.11	0.913	99.1 ± 5.2	102.8 ± 3.5	− 1.66	0.119
ABS/RC	Day 1	98.1 ± 9.5	102.7 ± 6.9	− 1.12	0.283	98.5 ± 12.6	95.2 ± 9.0	0.59	0.563
	Day 2	103.9 ± 8.2	99.9 ± 9.3	0.93	0.370	104.6 ± 15.5	96.0 ± 10.9	1.29	0.220
DI_0_/RC	Day 1	106.9 ± 27.3	104.3 ± 15.0	0.24	0.813	98.8 ± 18.3	82.9 ± 12.8	2.01	0.064
	Day 2	104.2 ± 17.9	100.7 ± 10.9	0.48	0.641	110.3 ± 36.5	86.5 ± 17.4	1.66	0.118
TR_0_/RC	Day 1	95.9 ± 6.4	102.3 ± 6.9	− 1.93	0.074	98.4 ± 12.2	99.5 ± 8.5	− 0.21	0.840
	Day 2	103.9 ± 7.0	99.7 ± 9.2	1.03	0.323	103.1 ± 11.1	99.0 ± 10.6	0.77	0.453
ET_o_/RC	Day 1	**86.7 ± 4.9**	**103.2 ± 9.5**	− **4.37**	**0.001**	**91.3 ± 6.5**	**117.4 ± 13.2**	− **5.03**	** < 0.001**
	Day 2	100.4 ± 8.4	99.7 ± 6.2	0.17	0.864	105.6 ± 10.4	98.5 ± 4.3	1.79	0.095
Phi (P_0_)	Day 1	98.0 ± 4.3	99.7 ± 3.0	− 0.92	0.376	**100.0 ± 2.8**	**104.2 ± 2.4**	− **3.26**	**0.006**
	Day 2	99.9 ± 3.1	99.8 ± 1.2	0.11	0.910	99.1 ± 5.2	102.8 ± 3.5	− 1.66	0.118
Psi (E_0_)	Day 1	**90.7 ± 8.5**	**100.8 ± 8.7**	− **2.36**	**0.033**	**92.7 ± 7.4**	**118.7 ± 14.3**	− **4.56**	** < 0.001**
	Day 2	96.6 ± 7.8	100.1 ± 13.5	− 0.64	0.535	104.1 ± 7.6	99.1 ± 8.5	1.26	0.230
Phi (E_0_)	Day 1	**89.1 ± 11.3**	**100.5 ± 8.6**	− **2.28**	**0.039**	**92.9 ± 7.3**	**120.9 ± 15.5**	− **4.61**	** < 0.001**
	Day 2	96.5 ± 8.6	99.9 ± 13.9	− 0.59	0.567	102.7 ± 11.2	101.6 ± 9.1	0.22	0.828
Phi (R_0_)	Day 1	**75.4 ± 10.2**	**100.3 ± 18.6**	** − 3.33**	**0.005**	104.0 ± 17.8	119.6 ± 77.2	− 0.56	0.587
	Day 2	88.1 ± 13.2	103.1 ± 26.2	− 1.44	0.171	95.6 ± 14.5	106.0 ± 18.0	− 1.27	0.226
ABS/CS	Day 1	115.9 ± 25.9	102.2 ± 21.3	1.16	0.265	**87.7 ± 16.1**	**113.1 ± 13.6**	− **3.42**	**0.004**
	Day 2	106.3 ± 17.7	102.8 ± 15.6	0.42	0.680	91.0 ± 16.1	88.8 ± 6.8	0.35	0.732
DI_0_/CS	Day 1	129.5 ± 51.9	103.8 ± 29.4	1.22	0.244	86.2 ± 22.1	92.3 ± 18.0	− 0.61	0.549
	Day 2	106.7 ± 23.7	103.5 ± 15.2	0.32	0.753	92.1 ± 26.9	80.3 ± 14.0	1.10	0.289
TR_0_/CS	Day 1	112.6 ± 19.9	101.7 ± 20.1	1.09	0.295	**88.1 ± 15.1**	**121.0 ± 12.2**	− **4.78**	** < 0.001**
	Day 2	106.2 ± 17.4	102.6 ± 16.0	0.43	0.673	90.7 ± 15.6	91.4 ± 6.6	− 0.12	0.904
ET_0_/CS	Day 1	101.2 ± 13.7	102.7 ± 25.5	− 0.15	0.883	**80.1 ± 18.5**	**159.8 ± 24.0**	− **7.43**	** < 0.001**
	Day 2	103.0 ± 25.8	102.6 ± 27.9	0.03	0.978	100.3 ± 20.3	89.4 ± 12.3	1.30	0.213
PI_ABS_	Day 1	84.7 ± 29.2	98.0 ± 20.3	− 1.06	0.309	**91.5 ± 26.1**	**135.3 ± 41.3**	− **2.54**	**0.024**
	Day 2	91.5 ± 26.7	99.4 ± 37.4	− 0.48	0.635	97.1 ± 35.5	109.7 ± 33.3	− 0.73	0.476

The results of Student's t-tests are provided in the table. Significant differences (*P* < 0.05) between the effect of salt and sucrose treatments are provided in bold

### Fluorescence kinetics: OJIP test

Regarding the first stage of the experiment, a reduced value of minimal fluorescence intensity (*F*_*0*_) was observed in most of the experimental groups after salt treatment compared to the control on both days of the experiment in the case of *H. physodes* (Fig. S5). A reverse tendency was observed for *P. furfuracea*, where after treatment with salt solutions the *F*_*0*_ values were higher in most experimental groups compared to the control (Fig. S6). As regards *H. physodes* treated with different concentrations of salt solutions, the fluorescence curves had a different course. On the first day of the experiment, for the concentrations of 3.5 M and 3.9 M, a clear decrease in the *F*_*M*_ value was visible. Moreover, the emission peak was not clearly marked and the induction curves of chlorophyll fluorescence were almost flat (Fig. S5). On the second day of the the experiment, a slight increase in the *F*_*M*_ value in these experimental groups can be observed. Chlorophyll fluorescence induction curves for *H. physodes* treated with sucrose solutions did not show significant deviations in relation to the control group, only a slight decrease in the *F*_*M*_ value was observed in single experimental groups compared to the control on the first day of the experiment. On the second day of experiment, the course of the chlorophyll induction curves was very similar in all experimental groups (Fig. S5). In the case of *P. furfuracea* thalli treated with salt on the first day of the experiment, a reduced value of the *F*_*M*_ was observed compared to the control, except for 0.9 M concentration (Fig. S6). After treatment of lichens with sucrose solutions, both on the first and the second day of the experiment, the *F*_*0*_ values were higher than in the control group. On the first day of the experiment, most of the chlorophyll induction curves ran considerably higher than in the control, with the exception of the groups representing the concentrations of 1.8 M and 2.8 M, for which the course was similar to the control group (Fig. S6).

**Fig. 2:**
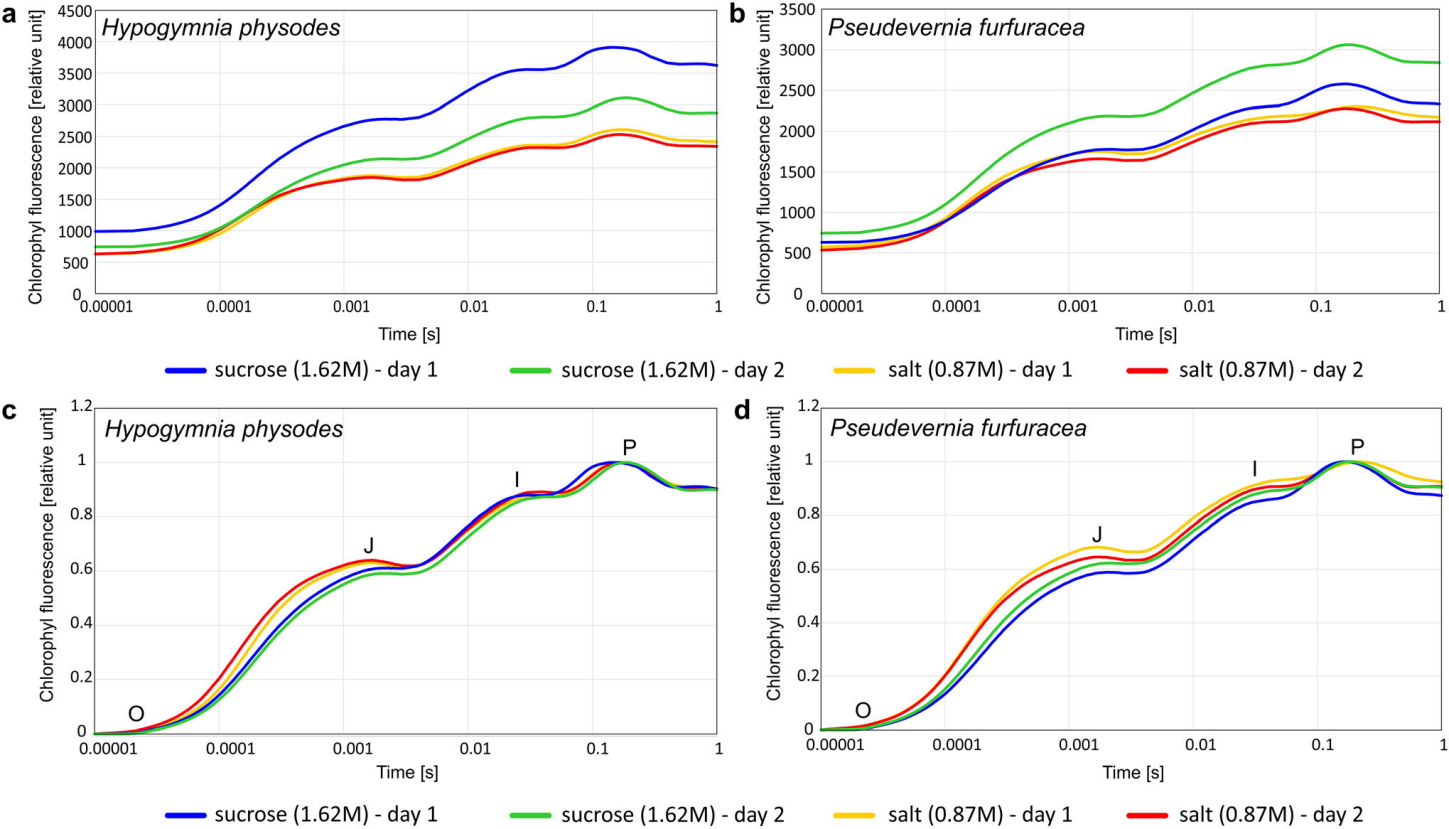
Chlorophyll fluorescence induction curves (log time scale) for *Hypogymnia physodes* (**a, c**) and *Pseudevernia furfuracea* (**b, d**) treated with salt solution (0.87 M) and sucrose solution (1.62 M) characterised by the same osmotic pressure (π = 39504 hPa) on the first and the second day of the experiment. The transient curves (**c, d**) are double normalised to *F*_*0*_ and *F*_*M*_

As regards the second stage of the experiment, the comparison of the effect of salt and sucrose solutions of the same osmotic pressure on the course of transient curves indicated that the curves had a flatter course and reached lower *F*_*M*_ values in the case of the salt solution compared to sucrose solution for both lichen species (Fig. 2a, b). The increased values at the O-J phase were observed in both lichen species treated with salt solution compared to sucrose solution on the first and the second day of the experiment (Fig. 2c, d).

## Discussion

### Effect of salt and sucrose stress on photosynthetic efficiency

The analysis of chlorophyll fluorescence is one of the most powerful and commonly utilised techniques to study the effect of stressful factors on the photosynthetic process. Many studies have investigated the effect of salt stress in different plant species (e.g. Kalaji et al. 2018; Zhao et al. 2019; Akhter et al. 2021); however, there are only a few studies that tested the effect of salt stress on lichens (e.g. Matthes-Sears et al. 1987; Nash III and Lange 1988; Smith and Gremmen 2001; Matos et al. 2011; Malaspina et al. 2015). Most of the previous studies analysed the issue in the context of lichens naturally exposed to sea salt, and even if they were experimental studies, they were concerned about the impact of seawater on lichen physiology. Moreover, the existing reports showed differentiated results in terms of the negative impact of salt on lichen photosynthesis. For example, Smith and Gremmen (2001) found that salinity did not significantly influence the CO_2_ assimilation rate in a shore-zone lichen *Turgidiusculum complicatulum*. On the contrary, incubation in a 100% artificial seawater solution of *Ramalina canariensis* caused a considerable and irreversible reduction in *F*_*V*_*/F*_*M*_ (Matos et al. 2011). The situation is more complicated since Nash III and Lange (1988) showed that the degree of sensitivity to salt stress of a certain species is proportional to the distance from the sea coast where the lichens grow. It can certainly be concluded that the sensitivity to salt stress is highly variable between species, but the response of species naturally occurring inland and exposed to salinity due to road salting remained poorly explored.

In regards to the photosynthetic process, osmotic stress can cause inactivation of PSII, photoinhibition, and electron flow retardation through PSII (Delmail et al. 2013); however, during dehydration, lichens can activate the specific mechanism for protection of PSII against ROS (Hajek et al. 2006). Osmotic stress leads to the loss of intracellular water, and when the water content is less than 10%, many enzymatic reactions and metabolism in cells are stopped (Green et al. 2011). The polyhydric nature of lichens makes them resistant to temporary lack of water availability, but ionic stress associated with the accumulation of ions in the cells has much more serious consequences especially when we compare these consequences for vascular plants, where even short-term drought stress itself can be fairly critical. Furthermore, in the case of vascular plants, the response to both salinity and drought stress proved to be similar at the early stage of *Tilia cordata* exposure to those stress factors and the response of PSII occurred even earlier in plants subjected to drought stress (Kalaji et al. 2018). Lichens are highly desiccation-tolerant organisms and most species can withstand drying to water contents of 5% or less remaining viable for a long time (Kranner et al. 2008). Based on our results, we can conclude that the short-term osmotic stress caused by sucrose does not cause considerable effects on photosynthetic efficiency in comparison with salt stress. This is in line with previous studies that showed that only prolonged staying in the desiccated state causes significant degradation of chlorophyll (Kranner et al. 2003; Beckett et al. 2005).

We have shown that the short-term salt stress leads to a significant reduction of *F*_*V*_*/F*_*M*_ which is a sensitive indicator of photosynthetic performance. This indicates photoinhibition of PSII, which is associated with damage to the reaction centres. Regarding the comparison between the studied lichen species, both the first and the second stage of the experiment confirmed that *H. physodes* is more sensitive to salt stress than *P. furfuracea*. Although both species involve photobionts of the genus *Trebouxia* and are typical inland species, one of them turned out to be more sensitive to salt stress. The greater sensitivity of *H. physodes* was evidenced not only by the greater decrease in the *F*_*V*_*/F*_*M*_ values but also by greater changes in chlorophyll fluorescence parameters and stronger deviation in the OJIP transient curve compared to the control. The high resistance *P. furfuracea* is in line with previous results of the study in which *P. furfuracea* proved to be more sensitive to atmospheric pollution than to the proximity to the sea (Malaspina et al. 2015). Sensitivity to stress may vary significantly between lichen species. Matos et al. (2011) reported that the incubation in 100% artificial seawater reduced *F*_*V*_*/F*_*M*_ in *Ramalina canariensis* even after 5 min of incubation. Moreover, subsequent incubation of the samples in deionized water did not recover *F*_*V*_*/F*_*M*_ to control values (Matos et al. 2011). Interestingly, lichen photobiont *Halofilum ramosum*, which is known so far only from marine environments, showed no clear reduction in *F*_*V*_*/F*_*M*_ in relation to NaCl concentration (Gasulla et al. 2019). Although there are several studies on the effect of salt stress that have analysed changes in photosynthetic efficiency over time, verifying the effect of multiple stress episodes over a longer period of time along with photosynthetic efficiency screening requires more in-depth studies. This would help to reflect the actual conditions to which lichens growing along salted communication routes are exposed and this issue is the subject of our further studies. The present study showed that exposure to short-term salt stress reduces the maximum quantum yield of PSII photochemistry, but does not cause permanent changes, because after 24 h from exposure to stress, *F*_*V*_*/F*_*M*_ values increased and in most experimental groups returned to the level characteristic of healthy lichens.

Another factor that affects the resistance of lichens to salt stress is the photobiont identity present in the thallus. The study on isolated *Trebouxia* sp. TR9 photobiont showed that this strain displays a superior performance under high salt concentrations and prolonged exposure did not significantly affect PSII activity and chlorophyll contents after a period of 72 h (Hinojosa-Vidal et al. 2018). However, it should be borne in mind that the isolated photobionts may react differently than those present in the lichen thallus. Kosugi et al. (2009) found different responses to dehydration between isolated *Trebouxia* sp. cells and those present in lichen *Ramalina yasudae*, the former of which were more sensitive to photoinhibition. This indicates that symbiotic association increases tolerance of photobiont (Kosugi et al. 2009).

Our results showed that compared to salt stress, the sucrosetreated lichen samples showed no significant reduction of the *F*_*V*_*/F*_*M*_ parameter even at high concentrations. Hajek et al. (2006) found that increasing sucrose concentrations and time of exposure led to a pronounced decrease in *F*_*V*_*/F*_*M*_ in *Lasallia pustulata* and *Umbilicaria hirsuta*; however, full loss of functioning primary photochemistry in PS II was observed after treatment with 2.5 M sucrose for 24 h. This could explain the lack of a significant response of the studied species to short-term sucrose stress.

### Fluorescence kinetics and selected parameters of fluorescence

OJIP fluorescence rise shape follows from changes in photosynthetic electron transport and the curve shows the successive reduction of electron transport pool of PSII (Govindjee 1995; Strasser et al. 2004). The trajectory of the curve provides information about the structure and functioning of the photosynthetic apparatus (Schreiber et al. 1994) and the shape of the OJIP transient has been found to be very sensitive to various kinds of environmental stress (Strasser et al. 2004). Our results showed that salt stress induced marked changes in the course of the fluorescence curve especially in the case of *H. physodes* (Figs. 2 and S5). On the contrary, the short-term osmotic stress caused by sucrose had no significant effect. The increase in NaCl concentration caused the decrease in *F*_*M*_, and it could result from the inhibition of electron transport on the donor side of PSII. The important issue is the assessment of whether *F*_*V*_*/F*_*M*_ decline, observed in both lichen species under salt stress, was due to the increase in *F*_*0*_ or variations of other components. The increase in minimal fluorescence intensity observed in *P. furfuracea* under salt stress may be due to mechanisms similar to those in plants, in which it was proved to indicate destruction or malfunction of PSII reaction centre or disruption in electron transport for excitation of reaction centres (Bolhar-Nordenkampf et al. 1989). The decreased values of *F*_*M*_ observed under salt stress indicate the accumulation of inactive reaction centres at PSII (Kalaji et al. 2011) and can be connected with cyclic electron transport within and around PSII and decrease in efficiency of the water splitting enzyme complex (Aro et al. 1993). In the case of *P. furfuracea*, we observed both rise in *F*_*0*_ and a decrease in *F*_*M*_, which resulted in a decrease of *F*_*V*_*/F*_*M*_ values. On the other hand, the decrease in *F*_*V*_*/F*_*M*_ under salt stress in *H. physodes* was mainly associated with a decrease in both *F*_*M*_ and *F*_*0*_ values. This indicates the increase in the rate constant of non-radiative energy dissipation that leads to a decrease in both minimal fluorescence intensity and maximal fluorescence intensity at closed PSII traps (Kitajima and Butler 1975). In contrast, in the case of *P. furfuracea*, only a decrease in the rate constant for PSII photochemistry could be recognized, which led to a rise in *F*_*0*_. Our results showed that the short-term osmotic stress caused by sucrose did not cause such a significant effect on either *F*_*V*_*/F*_*M*_ or changes in the trajectory of the fluorescence curve. It is worth noting, however, that the same trend of the increased *F*_*0*_ values in the case of *P. furfuracea* and decreased in the case of *H. physodes* due to sucrose stress was observed immediately after incubation. The study on *Lasallia pustulata* and *Umbilicaria hirsuta* showed that osmotic stress induced by sucrose caused no change or increase in *F*_*0*_ at low sucrose concentrations, while high concentrations caused a dramatic decrease in *F*_*0*_ (Hajek et al. 2006). This could also support the fact that the response to both salt and sucrose stress was greater in *H. physodes*. The manner of the response certainly depends on the stress factor under consideration. With regard to lichens, the decrease in *F*_*0*_ was caused by light stress (Balarinová et al. 2014) desiccation (Bednaříková et al. 2020) and biocides (Vannini et al. 2018); whereas the increase in *F*_*0*_ was induced by polycyclic aromatic hydrocarbons (Kummerová et al. 2006).

The results showed that incubation of lichens in high salt concentrations lead to a considerable increase in specific absorption flux per reaction centre (ABS/RC) and dissipation energy flux per reaction centre (DI_0_/RC); whereas Phi (R_0_), ET_0_/CS, ET_0_/RC and PI_ABS_ were significantly decreased compared to the control. With regard to high salt concentrations, the changes of these parameters were significantly stronger than in the case of *F*_*V*_*/F*_*M*_ (Fig. S3). It can therefore be concluded that even if the *F*_*V*_*/F*_*M*_ values are decreased due to stress, they may still remain at a high level in the range characteristic for healthy lichens, and in such cases the changes in these parameters may be a better determinant of salt stress. Akhter et al. (2021) found that salt treatment caused changes in OJIP curves in both salt-tolerant and salt-sensitive *Hordeum vulgare* genotypes. In this case, photoinhibition of PSII was mainly due to enhanced inactive reaction centres, reduced specific absorption flux per reaction centre (ABS/RC), low electron transport flux per reaction centre (ET_0_/RC) and decreased primary photochemistry (Phi (P_0_), TR_0_/RC). The increase of ABS/RC has been frequently observed in plants under drought stress (Van Heerden et al. 2007; Mehta et al. 2010; Gomes et al. 2012). Such a phenomenon could be explained by inactivation of some PSII RCs or an increase in antenna size (Kalaji et al. 2016). Nevertheless, the response depends on the sensitivity of the species in question and so, for example, ABS/RC and TR_0_/RC parameters increased after 60 min of photoinhibitory treatment in *Usnea antarctica*, whereas in *U. aurantiaco-atra* decreased (Balarinová et al. 2014). The study on *Dermatocarpon polyphyllizum* showed that after desiccation TR_0_/RC and ABS/RC increased and this may indicate that the number of active reaction centres is limited by the dehydration process (Bednaříková et al. 2020). The observed elevated level of dissipation energy flux per reaction centre (DI_0_/RC) could be interpreted as an agent to protect PSII from photooxidative damage in the thylakoids membrane (Akhter et al. 2021). Both lichen species showed a much slower electron transport flux per excited cross-section (ET_0_/CS) than the control samples under salt stress conditions. This could be associated with the inactivation of the reaction centre complex as it was observed in *Triticum aestivum* leaves after the treatment with various NaCl concentrations (Mehta et al. 2010). Malaspina et al. (2014b) obtained similar results after exposure of *P. furfuracea* to shady or light conditions. They reported a reduction of ET_0_/CS and Phi (E_0_) in light samples compared to shady ones which suggested malfunction of electron transport. They also observed that *F*_*0*_ and ABS/CS are late indicators of stress since differences between experimental groups were visible until after four weeks. The decrease of Phi (E_0_), Psi (E_0_) and ET_0_/RC with the simultaneous increase in DI_0_/RC was also observed due to the temperature drop in the lichen *Dermatocarpon polyphyllizum* (Marečková et al. 2019).

The PI_ABS_ constitutes a global indicator of the photosynthetic performance and indicates the overall vitality of the samples (Strasser et al. 2000). This parameter merges three functional steps of the photosynthetic activity (light absorption, excitation energy trapping, and electron transport), thus regarded as a very sensitive indicator of various kinds of stress in ecophysiological and environmental studies (Strasser et al. 2004). Our results also confirmed a stronger decrease of this parameter compared to *F*_*V*_*/F*_*M*_ due to salt stress. Similar observations were made in *Evernia prunastri* and *P. furfuracea* exposed to NH_3_ from livestock husbandry (Paoli et al. 2010). Nevertheless, another type of stress factor such as light stress can induce in lichens different effects depending on the species. For example, after 60 min of photoinhibition PI_ABS_ values increased in *U. aurantiaco-atra*, but the same treatment in *U. antarctica* resulted in a decrease of PI_ABS_ (Balarinová et al. 2014). On the contrary, the study on *Pleurosticta acetabulum* showed that extreme UVB radiation did not cause significant differences between experimental and control samples (Kyriatzi et al. 2021).

The maximum quantum yield of PSII photochemistry is one of the most frequently measured parameters in studies dealing with the influence of stress factors on plants and lichens. However, the results presented in this study prove that the values of other fluorescence parameters, e.g. those determining the electron transport efficiency or the minimal fluorescence intensity, can provide a lot of additional information about the physiological state of the photosynthetic apparatus. In the case of many parameters, the changes caused by salt stress were stronger than the changes in the *F*_*V*_*/F*_*M*_ parameter. Therefore, these parameters may be better indicators of the impact of salt stress on lichens and should be analysed in further studies.

### Ionic and osmotic effects of salt stress

Salt stress includes both osmotic stress and ionic toxicity (Mehta et al. 2010); however, it is difficult to assess unequivocally the relative importance of these two mechanisms as they overlap. Apart from osmotic stress induced by incubation of lichen thalli in the salt solution, a rapid uptake of ions to both intracellular and extracellular fractions occurs (Matos et al. 2011). This highlights the importance of determining the cellular location of elements in the context of studying the influence of ionic stress on lichen physiology. The results of the present study showed that solutions of salt and sucrose with the same osmotic pressure have a different effect on photosynthetic efficiency. The experiment showed that salt stress had a more negative impact on photosynthetic efficiency in comparison to control values than that of sucrose, which directly suggests additional negative effects of Na^+^ and Cl^−^ on photosynthesis. In the case of *P. furfuracea*, many fluorescence parameters can be considered as early indicators of salt-induced changes however no drastic reduction was observed in the *F*_*V*_*/F*_*M*_ as compared to the effects caused by salt and sucrose solutions with the same osmotic pressure. It concerns the decrease in the transfer of electrons per reaction centre and quantum yield of electron transport. It may also indicate the first effects be related to the impact of the ionic component of salt stress. The most symptomatic change was observed as the significant reduction in ET_0_/RC after treatment with salt solutions compared to both the control sucrose solution with the same osmotic pressure. This indicates a significant reduction of the utilisation of trapped energy in electron transport and thereby down-regulation of electron transfer. *Hypogymnia physodes* was characterised by a stronger response, since the studied parameters  including *F*_*V*_*/F*_*M*_ and PI_ABS_ were found significantly reduced due to osmotic stress induced by ionic (NaCl) solution compared to non-ionic (sucrose) solution.

Moreover, the course of fluorescence induction curves for salt and sugar treatments were significantly different. Raw OJIP transients showed that lichen samples subjected to salt stress had a decreased fluorescence mainly at I and P steps compared to samples treated with sucrose solution. The greater reduction in the fluorescence emission at these steps was also reported in different *Hordeum vulgare* genotypes subjected to salinity stress (Akhter et al. 2021). The double normalised transient curves showed that the main differences are associated with the O-J rise, whereas a significantly faster increase was observed in the case of lichens treated with salt solution compared to sucrose. The O-J phase corresponds to the photochemical reduction of the primary quinone electron acceptor of PS II (Q_A_) and accumulation of Q_A_^−^ (Strasser and Govindjee 1992; Kalaji et al. 2016). Consequently, a growth in this phase could indicate the reduction of PSII acceptor (Q_A_) (Boisvert et al. 2006). The prominent decrease in chlorophyll fluorescence signal and more flattened OJIP curve induced by salt stress was also found in freshwater algae and cyanobacteria (Zhang et al. 2010; Vilumbrales et al. 2013).

## Conclusions

Our results show that the short-term salt stress leads to a significant reduction of *F*_*V*_*/F*_*M*_ and greater changes in chlorophyll fluorescence parameters and OJIP transient curve compared to the osmotic stress induced by sucrose. This proved that salt stress is associated not only with the osmotic stress that causes physiological drought by cell dehydration but also with negative effects of excessive accumulation of ions in cells (ionic stress). The most symptomatic effect of the ionic stress observed a significant reduction in the utilisation of trapped energy in electron transport and thereby down-regulation of electron transfer. In the case of lichens, which are poikilohydric organisms resistant to temporary lack of water availability, ionic stress associated with accumulation of ions in the cells have much more serious consequences especially when these consequences were compared with vascular plants vulnerable to drought stress.

Both the first and the second stage of the experiment showed that *H. physodes* is more sensitive to salt stress than *P. furfuracea*. The greater sensitivity of *H. physodes* to salt stress was evidenced not only by stronger decrease in *F*_*V*_*/F*_*M*_ values, but also by greater changes in chlorophyll fluorescence parameters and stronger deviation in OJIP transient curve compared to the control treatment.

The maximum quantum yield of PSII photochemistry is one of the most widely studied parameters describing photosynthetic performance of plants and lichens exposed to stress factors. However, the results showed that changes of other fluorescence parameters could be more informative and provide a lot of additional information about the physiological state of the photosynthetic apparatus. In the case of many parameters, the changes caused by salt stress were stronger than the changes in the *F*_*V*_*/F*_*M*_ parameter compared to control. Consequently, these parameters could be better indicators of the impact of salt stress on lichens.

The results showed that exposure to short-term salt stress reduces the photosynthetic efficiency, but does not cause permanent changes, because after 24 h from exposure to stress, *F*_*V*_*/F*_*M*_ values increased and in most of experimental groups returned to the level characteristic for healthy lichens. Nevertheless, short-term but repeated episodes of exposure to salt stress may reduce the vitality of lichens inhabiting the substrates along communication routes which may result in a consequence to the death of the lichen thallus. In many countries the period of salt sprinkling on roads is very long, so it is important to look for alternatives to road salt with a less harmful impact on the surrounding environment.

## Supplementary Information

Below is the link to the electronic supplementary material.

Supplementary file1 (PDF 2612 kb)
