# Supplementary material for: Evaluation of the importance of ionic and osmotic components of salt stress on the photosynthetic efficiency of epiphytic lichens
Source: Physiol Mol Biol Plants. 2022 Feb 3;28(1):107–21. doi: 10.1007/s12298-022-01134-2 (PMC8847468; doi:10.1007/s12298-022-01134-2)
Supplement: Supplementary file 1 — Supplementary file1 (PDF 2612 kb) [file 12298_2022_1134_MOESM1_ESM.pdf]

SUPPLEMENTARY MATERIALS TO THE ARTICLE

Evaluation of the importance of ionic and osmotic components of salt stress on the photosynthetic efficiency of epiphytic lichens

Karolina Chowaniec[1], Kaja Rola [1]*

[1] Institute of Botany, Faculty of Biology, Jagiellonian University, Gronostajowa 3, 30-387 Kraków, Poland

* Corresponding author. E-mail address: kaja.skubala@uj.edu.pl (K. Rola)

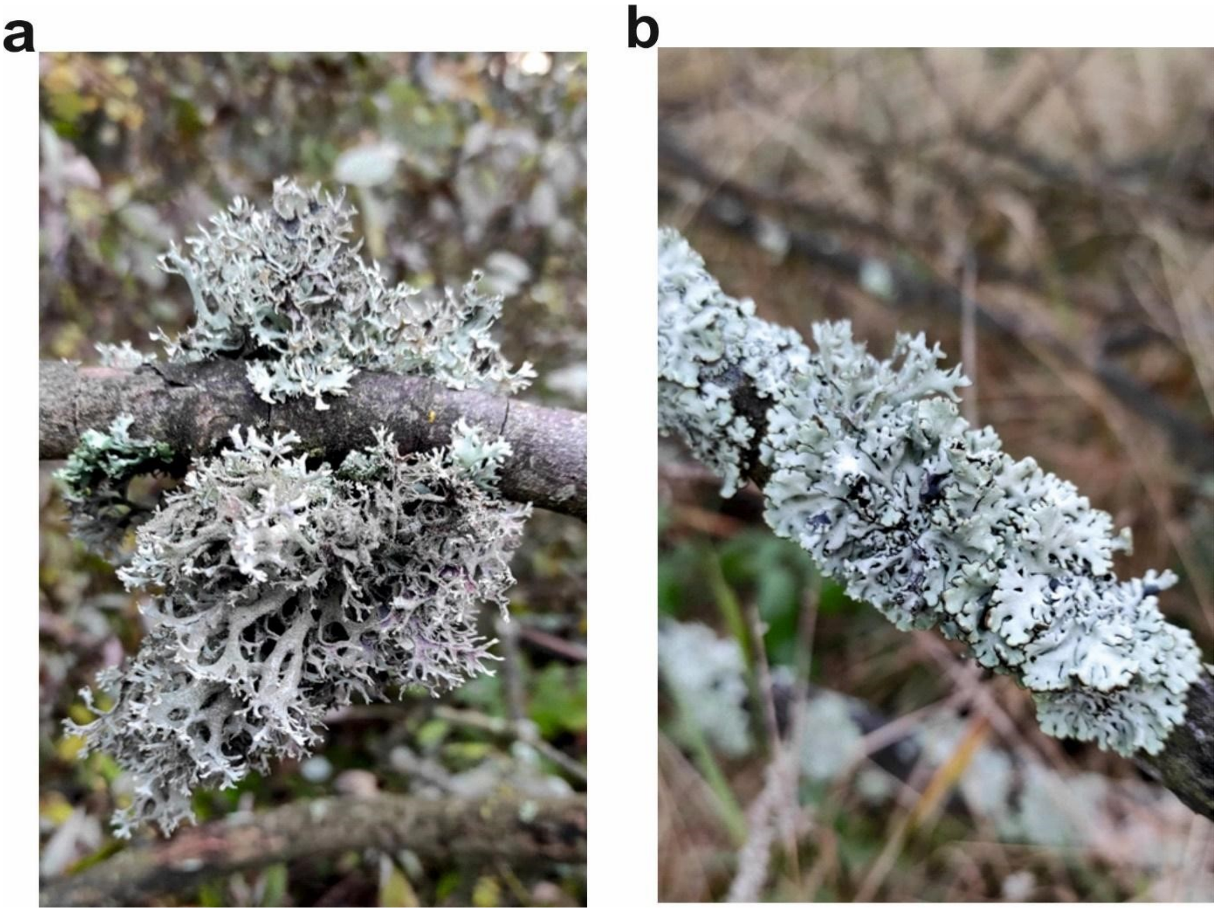

Fig. S1 Lichen species analysed during the study: *Pseudevernia furfuracea* (**a**) and *Hypogymnia physodes* (**b**)

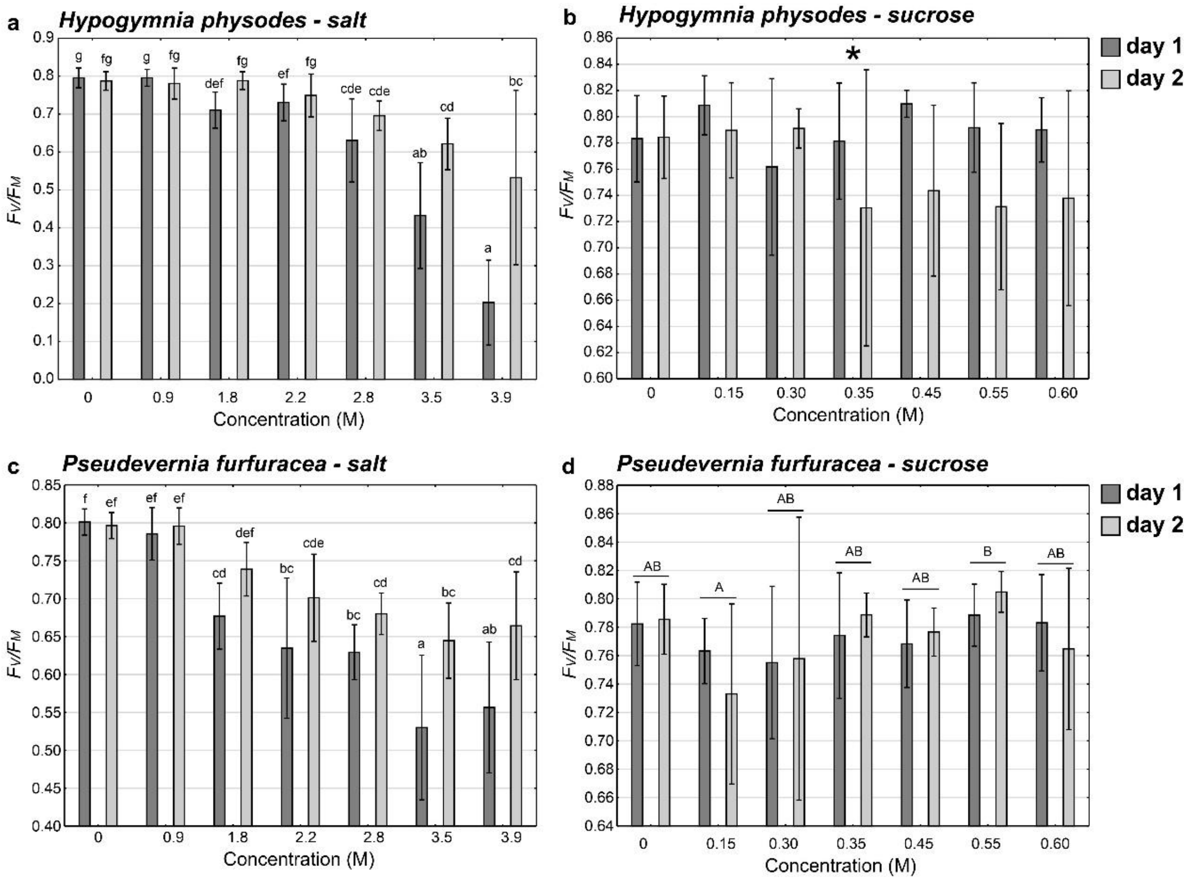

Fig. S2 The F_V/F_M parameter in particular experimental groups (means ± SE; n = 8) for different concentrations of salt and sucrose solutions in two subsequent days of the experiment for *Hypogymnia physodes* (**a, b**) and *Pseudevernia furfuracea* (**c, d**). The different letters above the bars indicate statistically significant differences (P < 0.05). Lowercase letters indicate statistically significant interaction between day of the experiment and solution concentration. Capital letters above the bars show the significant main effect of solution concentration. The asterisk indicates the significant main effect of the day of the experiment; for each P < 0.05. See Table S1 for details on the main effects and interactions

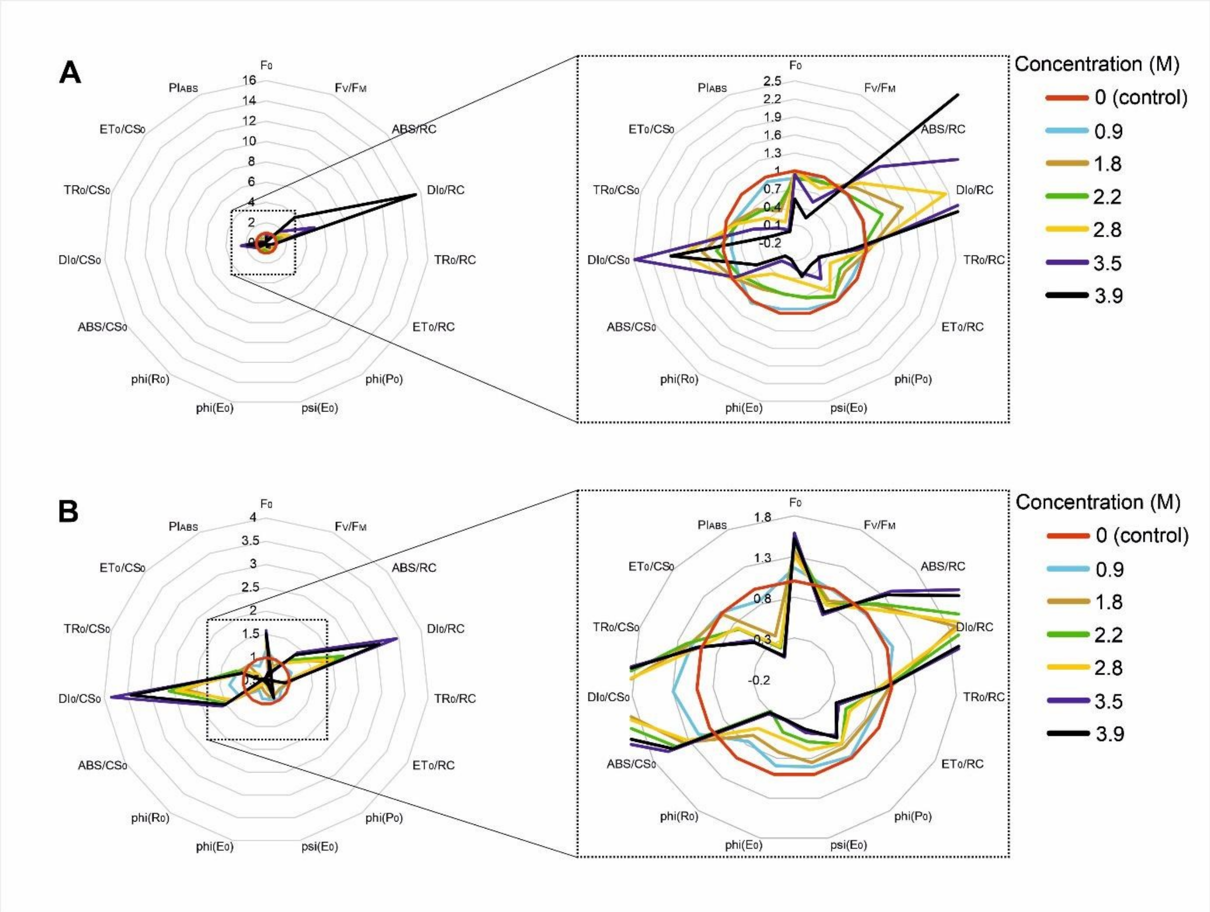

Fig. S3 The spider plots showing the effect of salt stress (solutions of different NaCl concentrations) on various photosynthetic parameters characterising PSII functionality on the first day of the experiment for *Hypogymnia physodes* (**a**) and *Pseudevernia furfuracea* (**b**). The plots are based on normalised values to the control treatment, enabling comparison of the variables measured on different scales

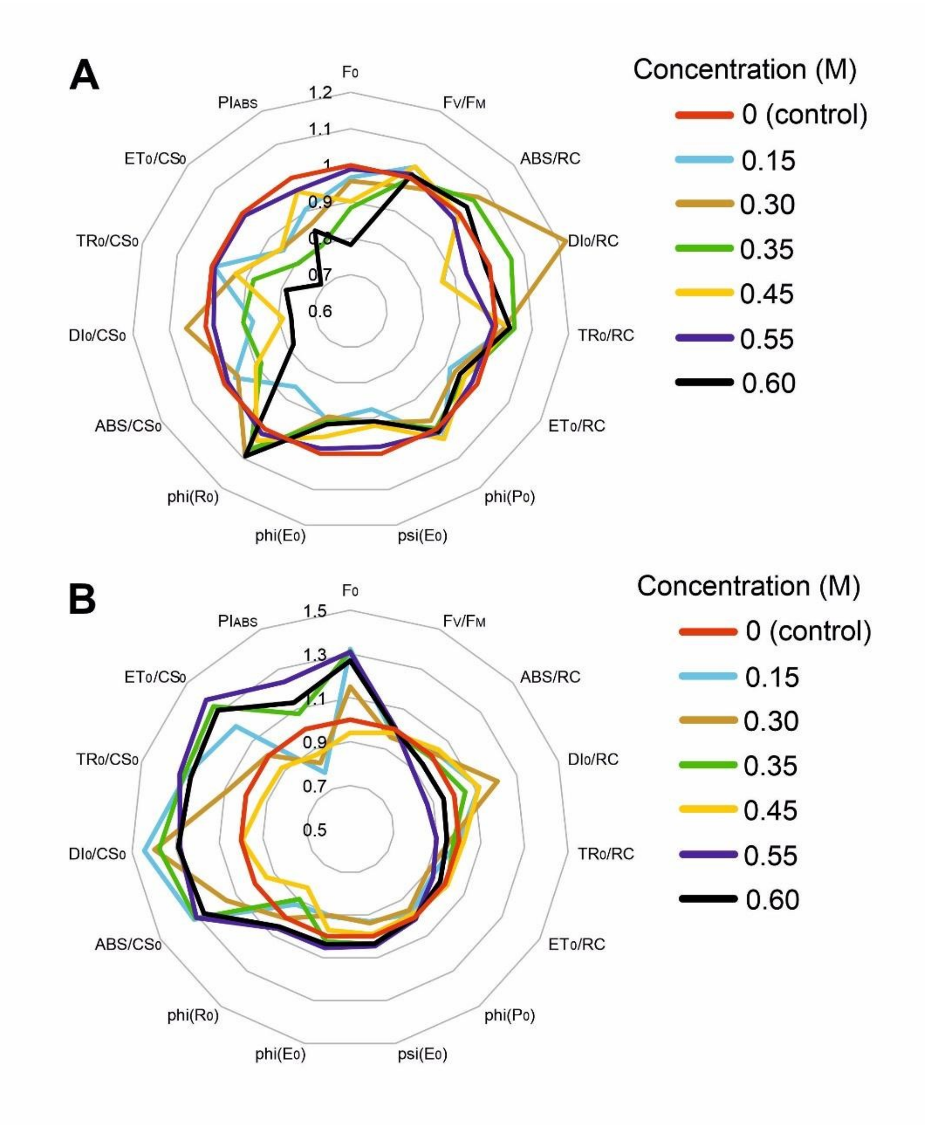

Fig. S4 The spider plots showing the effect of sucrose stress (solutions of different concentrations) on various photosynthetic parameters characterising PSII functionality on the first day of the experiment for *Hypogymnia physodes* (**a**) and *Pseudevernia furfuracea* (**b**). The plots are based on normalised values to the control treatment, enabling comparison of the variables measured on different scales

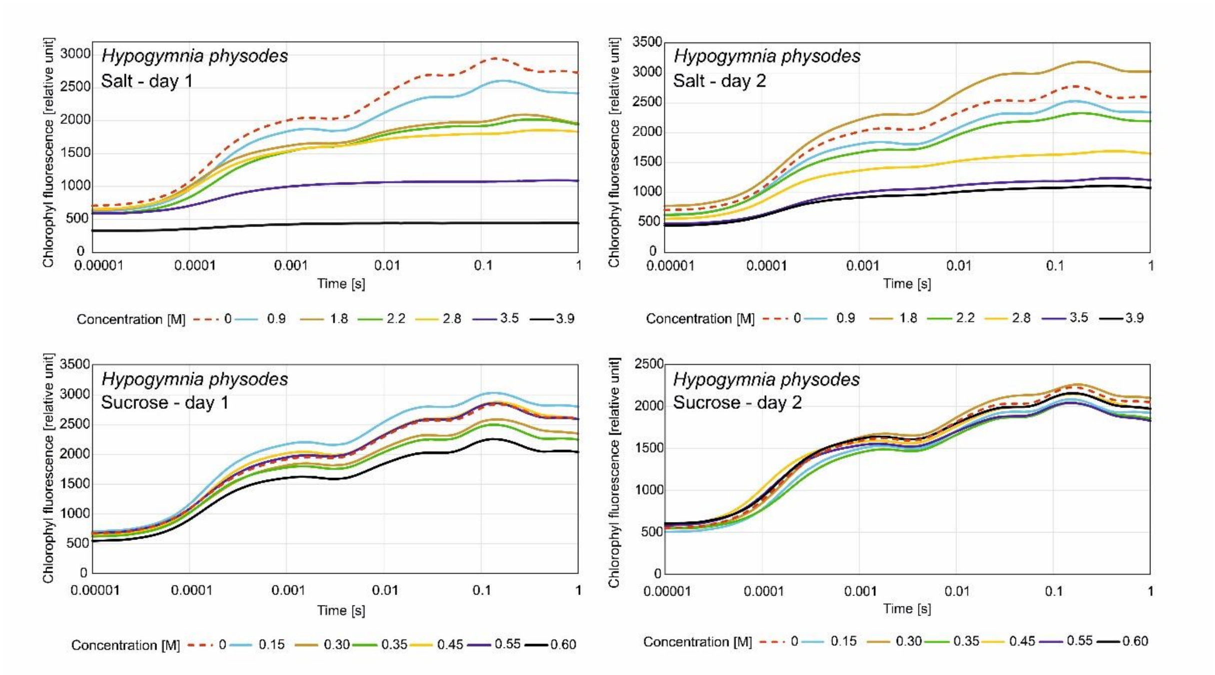

Fig. S5 Chlorophyll fluorescence induction curves for *Hypogymnia physodes* treated with different concentrations of salt and sucrose solutions on the first and the second day of the experiment

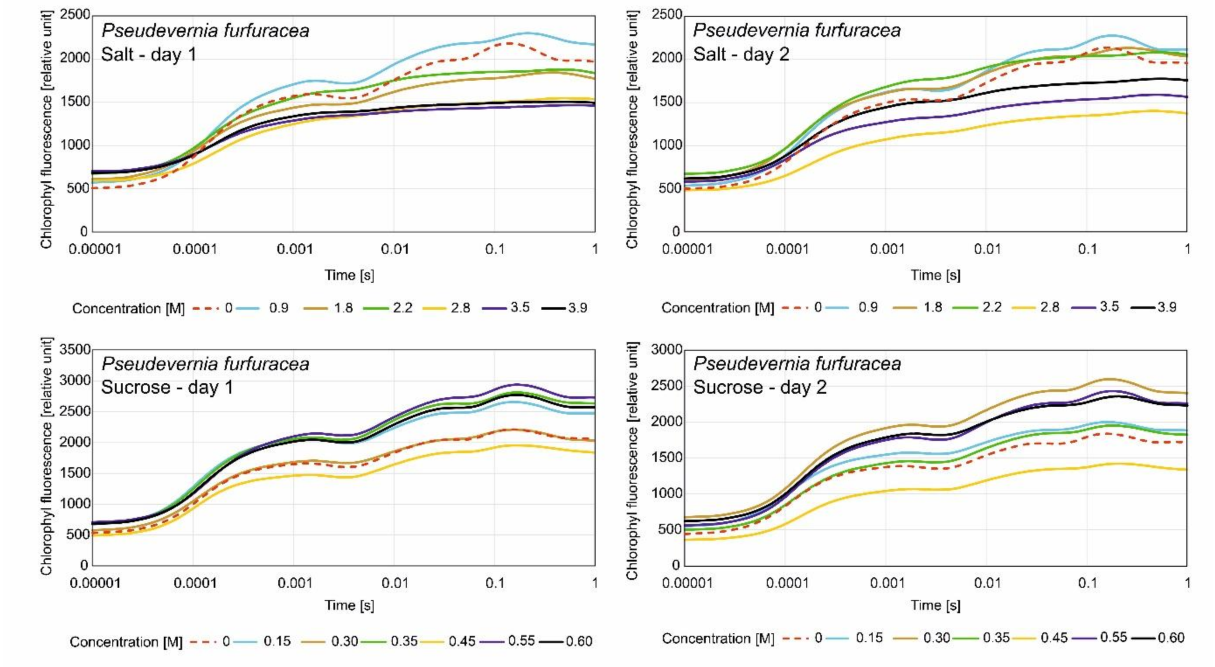

Fig. S6 Chlorophyll fluorescence induction curves for *Pseudevernia furfuracea* treated with different concentrations of salt and sucrose solutions on the first and the second day of the experiment

Table S1 Results of two-way analysis of variance for the effect of 'Day' and 'Concentration' after treatment with salt and sucrose solutions on the F_V/F_M parameter for *Hypogymnia physodes* and *Pseudevernia furfuracea* and for the effect of 'Species' and 'Concentration' after treatment with salt and sucrose solutions on the F_V/F_M parameter on the first and the second day of the experiment

Lichen species	*Hypogymnia physodes*										*Pseudevernia furfuracea*									
Independent variables	Day			Concentration			Day × Concentration			Error	Day			Concentration			Day × Concentration			Error
	F	P	df	F	P	df	F	P	df	df	F	P	df	F	P	df	F	P	df	df
Sucrose	10.12	0.002	1	1.26	0.284	6	1.80	0.107	6	98	0.14	0.705	1	2.67	0.019	6	0.69	0.655	6	98
Salt	28.28	<0.001	1	63.71	<0.001	6	5.08	<0.001	6	98	30.48	<0.001	1	35.04	<0.001	6	2.58	0.023	6	98
Day of experiment	Day 1										Day 2									
Independent variables	Species			Concentration			Species × Concentration			Error	Species			Concentration			Species × Concentration			Error
	F	P	df	F	P	df	F	P	df	df	F	P	df	F	P	df	F	P	df	df
Sucrose	7.25	0.008	1	1.10	0.366	6	1.33	0.250	6	98	1.76	0.188	1	0.72	0.637	6	2.93	0.011	6	98
Salt	10.03	0.002	1	68.17	<0.001	6	15.34	<0.001	6	98	0.39	0.535	1	38.71	<0.001	6	3.24	0.006	6	98

Table S2 Photosynthetic parameters characterising PSII functionality (means ± SE) in the species *Pseudevernia furfuracea* and *Hypogymnia physodes* after treatment with 0.87 M salt solution and 1.62 M sucrose solution characterised by the same osmotic pressures and control treatments for both experimental groups. The values in bold indicate the presence of significant differences between the salt/sucrose treatments and the control treatment according to Student's t-tests ($P < 0.05$)

Parameter	Day of experiment	*Pseudevernia furfuracea*				*Hypogymnia physodes*			
		Salt	Control	Sucrose	Control	Salt	Control	Sucrose	Control
F_0	Day 1	496.38±36.64	428.12±24.68	559.88±38.51	547.88±33.01	532±32.32	606.75±65.80	870.25±34.49	769.38±113.82
	Day 2	461±25.36	433.63±29.37	647±32.57	629.38±34.52	543.50±31.76	597.50±75.53	645.38±16.44	726.75±51.23
F_V/F_M	Day 1	0.79±0.01	0.80±0.01	0.78±0.01	0.78±0.01	0.80±0.01	0.80±0.01	0.78±0.01	0.75±0.02
	Day 2	0.80±0.01	0.80±0.01	0.79±0.00	0.79±0.00	0.78±0.01	0.79±0.01	0.79±0.01	0.77±0.01
ABS/RC	Day 1	3.22±0.10	3.28±0.06	2.80±0.06	2.73±0.07	3.14±0.13	3.19±0.12	2.86±0.09	3.01±0.13
	Day 2	3.22±0.08	3.10±0.08	2.89±0.09	2.89±0.09	3.39±0.17	3.24±0.11	2.75±0.10	2.86±0.17
DI_0/RC	Day 1	0.70±0.06	0.65±0.03	0.61±0.03	0.59±0.01	0.64±0.04	0.65±0.03	0.64±0.03	0.77±0.08
	Day 2	0.66±0.04	0.63±0.03	0.61±0.02	0.61±0.02	0.76±0.08	0.69±0.03	0.58±0.04	0.67±0.06
TR_0/RC	Day 1	2.52±0.06	2.63±0.05	2.19±0.05	2.14±0.06	2.50±0.10	2.54±0.10	2.22±0.06	2.24±0.10
	Day 2	2.56±0.06	2.47±0.05	2.28±0.07	2.28±0.07	2.63±0.09	2.55±0.09	2.17±0.08	2.19±0.11
ET_o/RC	Day 1	**0.78±0.01**	**0.90±0.02**	0.86±0.03	0.83±0.03	**0.88±0.02**	**0.96±0.03**	0.83±0.03	0.71±0.09
	Day 2	0.86±0.02	0.86±0.02	0.81±0.02	0.81±0.02	0.91±0.03	0.86±0.09	0.85±0.01	0.86±0.03
Phi (P_0)	Day 1	0.79±0.01	0.80±0.01	0.78±0.01	0.78±0.01	0.80±0.01	0.80±0.01	0.78±0.01	0.75±0.02
	Day 2	0.80±0.01	0.80±0.01	0.79±0.00	0.79±0.00	0.78±0.01	0.79±0.01	0.79±0.01	0.77±0.01
Psi (E_0)	Day 1	**0.31±0.01**	**0.34±0.01**	0.39±0.01	0.39±0.01	0.35±0.01	0.38±0.02	0.37±0.01	0.31±0.04
	Day 2	0.34±0.01	0.35±0.01	0.36±0.02	0.36±0.02	0.35±0.01	0.33±0.04	0.39±0.01	0.40±0.02
Phi (E_0)	Day 1	**0.24±0.01**	**0.27±0.01**	0.31±0.01	0.31±0.01	0.28±0.01	0.30±0.01	0.29±0.01	0.24±0.03
	Day 2	0.27±0.01	0.28±0.01	0.28±0.01	0.28±0.01	0.27±0.01	0.26±0.03	0.31±0.01	0.31±0.01
Phi (R_0)	Day 1	**0.07±0.00**	**0.09±0.00**	0.11±0.01	0.11±0.01	0.10±0.01	0.09±0.01	0.09±0.02	0.08±0.01
	Day 2	0.08±0.00	0.09±0.00	0.09±0.01	0.09±0.01	0.08±0.00	0.09±0.01	0.10±0.01	0.10±0.01
ABS/CS	Day 1	496.38±36.64	428.13±24.68	559.88±38.51	547.88±33.01	532±32.32	606.75±65.80	870.25±34.49	769.38±113.82
	Day 2	461±25.36	433.63±29.37	647±32.57	629.38±34.52	543.50±31.76	597.50±75.53	645.38±16.44	726.75±51.23

DI_0/CS	Day 1	109.31±14.48	84.43±4.06	122.90±11.49	118.37±8.27	109.65±9.29	127.27±20.19	194.77±12.53	210.95±53.58
	Day 2	94.45±6.94	88.49±7.03	136.79±6.64	132.12±7.15	120.02±11.59	130.34±21.67	135.44±7.84	168.76±15.30
TR_0/CS	Day 1	387.06±22.65	343.70±21.19	436.98±28.49	429.51±25.92	422.35±24.01	479.48±46.37	675.48±22.51	558.42±61.43
	Day 2	366.55±19.90	345.13±22.84	510.21±26.29	497.26±27.70	423.48±24.17	467.16±54.50	509.93±12.24	557.99±38.21
ET_0/CS	Day 1	118.85±5.31	117.42±7.61	173.88±14.28	169.23±12.90	150.40±11.48	187.67±27.60	252.10±12.53	157.79±22.27
	Day 2	124.95±10.37	121.36±9.32	185.22±16.64	180.57±17.19	146.82±9.80	146.33±20.40	200.94±9.15	224.75±23.16
PI_{ABS}	Day 1	0.55±0.06	0.65±0.04	0.85±0.06	0.87±0.05	0.71±0.07	0.77±0.05	0.76±0.08	0.56±0.09
	Day 2	0.64±0.06	0.70±0.06	0.75±0.09	0.76±0.09	0.61±0.07	0.63±0.09	0.94±0.09	0.85±0.13
